# Suicidal Ideation in Adolescence: A Perspective View on the Role of the Ventromedial Prefrontal Cortex

**DOI:** 10.3389/fpsyg.2020.00713

**Published:** 2020-04-15

**Authors:** Rosalba Morese, Claudio Longobardi

**Affiliations:** ^1^Faculty of Communication Sciences, Università della Svizzera Italiana, Lugano, Switzerland; ^2^Institute of Public Health, Faculty of Biomedical Sciences, Università della Svizzera Italiana, Lugano, Switzerland; ^3^Department of Psychology, University of Turin, Turin, Italy

**Keywords:** suicidal ideation, fMRI, adolescence, social exclusion, emotion regulation, ventromedial prefrontal cortex, anterior insula

## Abstract

Suicide in adolescence is a worldwide issue, and it continues to present a serious problem in terms of its prevention. Among the various aspects of suicide, a very interesting area of research is represented by suicidal ideation. Recently, neuroimaging-based methods have made it possible to study the cognitive processes involved in several social situations and clinical conditions. This theoretical perspective article with an interdisciplinary approach integrates evidence from developmental psychology and social neuroscience with the aim of investigating the role of the brain area responsible for regulating negative emotions during the cognitive processes of suicidal ideation: the ventromedial prefrontal cortex. By highlighting the role of brain areas in the few studies published so far, it is possible to develop perspectives of considerable impact. Studying and understanding the role of brain areas involved in suicidal thoughts in adolescents could facilitate the creation of new perspectives on prevention programs and interventions.

## Introduction

Worldwide, suicidal ideation (SI) is one of the two leading causes of death in adolescents in the 15–19-year-old age group (World Health Organization, 2014). The prevalence of suicide attempts in the United States is 4.1%, while, in Europe, the lifetime prevalence is similar (4.2%; Carli et al., 2014). SI is defined as “thoughts about death, dying, plans for suicide, or desire for death” (Miller et al., 2018; Levi-Belz et al., 2019). The theoretical perspective aims to review the Ventromedial Prefrontal Cortex’s role and its association with risk factors, such as anxiety, depression, and social isolation, involved in adolescents experiencing SI thoughts. This view could facilitate the creation of better and specific prevention programs and interventions supporting adolescents at high risk of SI. Unfortunately, to date, few studies have investigated this research topic using neuroimaging methodologies in the form of functional magnetic resonance imaging (fMRI; Groschwitz et al., 2016; Johnston et al., 2017). This lack is mainly due to the type of experimental sample required; the adolescent population is very difficult to recruit. Indeed, Miller et al. (2017) claimed that this is the principal reason why, to date, few studies have investigated and explored the neural correlates associated with the risk of SI.

## Ventromedial Prefrontal Cortex

The ventromedial prefrontal cortex (VMPFC) plays an important role in controlling social tasks, including moral judgment, social decision-making, and social emotions, as well as in regulating emotions (Koenigs et al., 2007). Hiser and Koenigs (2018), in a recent review, have deepened the main role of VMPFC in the various aspects of the emotional and social process of social cognition, such as in the representation of value in social decision-making and the regulation of emotions. In fact, through its connections with the ventral striatum and the amygdala, it is recruited in cognitive reward processes, but it is implicated, above all, in negative experiences as per the regulation processes of negative emotions. For this reason, patients with VMPFC lesions show a worsening in terms of social relationships (Beadle et al., 2018). The VMPFC function is given by its anatomical connections with other areas of the brain implicated in emotions (e.g., the amygdala that is important for the emotional recognition of fear and anger and the hippocampus and the anterior cingulate cortex, which are crucial in the inhibition of behavioral response). Furthermore, its regulatory function depends on its interface with the posterior cingulate cortex, the precuneus, the dorsomedial PFC, and the neural network involved during the representations of the theory of the mind, and on its ability to represent mental states, emotions, and beliefs about the self and other people (Premack and Woodruff, 1978). Traditionally, the brain circuit underlying the theory of the mind implicated the recruitment of the medial prefrontal cortex (MPFC) and temporal parietal junction (TPJ; Amodio and Frith, 2006; Frith and Frith, 2006). Both the medial and prefrontal cortices are located within the prefrontal cortex. The difference between them is in their connections, and only the VMPFC directly interfaces with the limbic structures of emotions. Therefore, the VMPFC can play a central role in regulating negative emotions, but its role in complex social situations is not yet sufficiently investigated as in the case of suicidal thoughts (Amodio and Frith, 2006; Frith and Frith, 2006). Koenigs and Grafman (2009) in a complete and critical review on the functional neuroanatomy of depression, discussed the VMPFC as critical neural substrates for depression. According to authors its impact may be due to self-awareness or self-reflection processes. But to date VMPFC role is not yet sufficiently investigated, not just for the depression but also for complex social situations as the suicidal thoughts (Amodio and Frith, 2006; Frith and Frith, 2006; Koenigs and Grafman, 2009).

Among the few studies in the literature, the recent research by Johnston et al. (2017) found very important results in adolescents who had attempted suicide several times. Using fMRI, they showed a reduced volume of gray matter in the brain areas of the cerebellum and the hippocampus, but, above all, they highlighted innovative results while adolescents observed facial emotional expressions. The fMRI results showed poor functional connectivity between the VMPFC and the amygdala, indicating a disconnection with negative emotions (Johnston et al., 2017).

Morese et al. (2016) revealed the recruitment of the VMPFC during the punishment of unfair behavior in in-group settings. Their study investigated the brain area involved during the decision-making related to punishment; in the opinion of the authors, the negative emotions experienced by participants may have been evoked by the inhibition of the VMPFC. Subsequently, Lo Gerfo et al. (2019) replicated this study with the application of a different neurophysiological method: anodal transcranial direct current stimulation (tDCS). The authors applied tDCS to modulate VMPFC activity during the punishment of different group members, and the sense of belonging was manipulated by cultural belonging (see Lo Gerfo et al., 2019 and Morese et al., 2016 for details). Lo Gerfo et al. (2019) found that the VMPFC is crucial for regulation processes in cooperation and altruistic behaviors for social interactions in in-group memberships. Concerning this aspect, the role of the ventromedial assumes great importance in social life, when people interact with others, especially within the social groups to which they belong. In particular, this aspect gains weight in the period of adolescence (Morese et al., 2020). In line with this theoretical framework, Morese et al. (2019a) underlined how Garner and Hinton (2010) found a negative correlation between emotional self-regulation and experiences of social exclusion, concerning emotions of anger and sadness, in a sample of adolescents. Indeed, self-regulation is a capacity for healthy development and functioning. This ability includes managing and modulating one’s own behavior and having good social skills and maintaining social interactions, while taking into account the feelings and needs of other people (Eisenberg et al., 2010).

## Suicidal Ideation and Social Exclusion

Recent studies (Malhi et al., 2019; Sommer et al., 2019) have investigated SI, but only a few studies have used fMRI. In this regard, Oppenheimer et al. (2019) conducted a very important study on adolescent SI, considering the impact of social exclusion as a facilitating factor for suicide. In their study, adolescents with high levels of anxiety were recruited and underwent the experimental condition of social exclusion. Again, Oppenheimer et al. (2019) examined whether this kind of social interaction can modulate suicidal thoughts. It was the first study that has investigated the neural response to a negative external condition, defining it as a factor that can promote SI. The results of their research showed that adolescents with higher levels of anxiety reported greater SI after experiencing social exclusion (Oppenheimer et al., 2019). Greater activation of the right anterior insula (AI) was found; it represents the principal brain correlate of social exclusion networks (Eisenberger et al., 2003). In addition, other important results from this research indicated that participants reported more negative experiences and victimization from peers in their daily lives. Therefore, the authors assumed that the experience of social exclusion could favor SI because these brain areas are very sensitive to this kind of vulnerability (Oppenheimer et al., 2019). As further evidence of this, a recent study (Oliè et al., 2017) has shown that in women who have committed suicide attempts, a reduced activation of the insula during experiences of social exclusion was observed. The literature reported above suggests that the experience of social exclusion may favor the greater activation of the brain structures of social exclusion with a greater involvement of the VMPFC and, therefore, also a greater possibility of developing SI.

## Suicidal Ideation and Emotion Regulation

**FIGURE 1:**
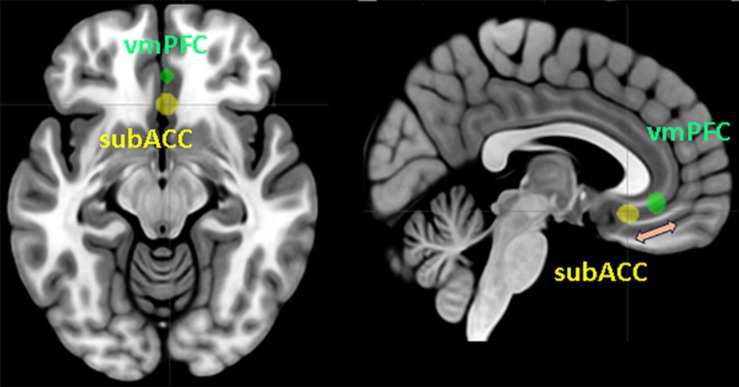
Ventromedial prefrontal cortex (VMPFC) and subgenual region of the anterior cingulate cortex (subACC) superimposed on a standard T1 template. The figure represents the modulation between the two brain areas in the process of emotion regulation. Spheres with a 5 mm radius centered on the Montreal Neurological Institute (MNI) coordinates reported by Morese et al. (2019a) were generated using the MarsBaR toolbox (Brett et al., 2002). The MRIcron software package (Rorden et al., 2007) was applied for sphere display.

Miller et al. (2018), in their study, underlined how some previous research showed that a poor ability to regulate emotions is related to SI, but they examine it in detail for the first time. They recruited adolescents with and without SI, to whom they showed negative emotional images and then an emotional regulation task. Their results are very important because they show that no difference emerges between the groups in the observation of negative images, but a significant difference occurs in the emotion regulation task. The sample of adolescents with SI reported a minor activation of the TPJ and dorsolateral prefrontal cortex (DLPFC) areas. The deactivation of these areas implies a greater activation of the AI and the anterior cingulate cortex, the brain network relating to social exclusion, which is responsible for negative emotions. These results are in line with recent research (Morese et al., 2019b) about the modulation of social support on the brain area related to social exclusion. The authors found that the recruitment of TPJ modulates the negative emotions experienced during social exclusion, and when this area is deactivated by informative messages, it understands the negative social situation of exclusion, and negative emotions increase during the next negative experience. Concerning this area, it seems to have the function of modulating the VMPFC through the representation of what is happening in terms of negative emotions in very difficult situations, such as during an experience of social exclusion. The increase in negative emotions seems to be related to a greater activation of the subgenual region of the anterior cingulate cortex (subACC). Therefore, difficulty in emotion regulation could facilitate the greater involvement of this area in depressed patients. This could suggest the modulating role of the ventromedial brain area on the activation of the subACC (Figure 1).

In this regard, another study explores how social rejection can represent a potential risk factor for depressed adolescents with non-suicidal self-injury (NSSI; Groschwitz et al., 2016). The authors investigated the neural correlates of social exclusion in 28 depressed adolescents: 14 with NSSI, 14 without NSSI, and 15 who were healthy. The fMRI results show that NSSI-depressed adolescents activate the ventrolateral prefrontal cortex (VLPFC) and the MPFC more than depressed adolescents without NSSI. This neurophysiological evidence suggests that the factors that predispose people to social exclusion are important points for planning interventions and social inclusion programs regarding emotion regulation in adolescents.

## Discussion

Social exclusion and emotion dysregulation related to anxiety and depression in adolescents are the most important predictive factors of SI or suicide attempts. Interventions that aim at preventing SI in adolescents before such thoughts develop into suicidal behavior are very important (O’Connor and Nock, 2014). The need to belong is postulated, from the perspective of self-determination theory (Deci and Ryan, 1985), as one of the three basic psychological needs that are the basis of self-regulation and psychological well-being. Individuals may be particularly sensitive to this need during adolescence, considering that this is when the peer group becomes a primary social reference with respect to the family (Somerville, 2013; Marengo et al., 2018; Badenes-Ribera et al., 2019). A thwarted sense of belonging may affect people’s psychological well-being, increasing emotional distress and dysregulation (Marengo et al., 2018; Settanni et al., 2018). By nature, social exclusion can counteract the sense of belonging in adolescents (Cheek et al., 2020). Several theories, including the interpersonal theory of suicide (ITS; Joiner, 2005) or the three-step theory (Klonsky and May, 2015), postulate a link between social exclusion and the increasing risk of suicidal thoughts and behavior (Cheek et al., 2020). In accordance with pain overlap theory (Panksepp, 1998), social and physical pain share common neural systems. Social exclusion, thwarting interpersonal needs, may result in social pain, which promotes an activation of the physical pain system. Suicide can be a form of response to mental pain. In particular, the role of neuroscience can help highlight other aspects as well as those that are already well known. Neuroscience research can investigate the functioning of neurophysiological correlates characterized by SI. This can help in the planning and designing of interventions that are useful for preventing suicide in adolescence. Better understanding the role of the VMPFC in the regulation of emotions can help in planning educational and social interventions that are useful for the development of the emotional component of adolescents and for decreasing risk and promoting health. A deeper understanding of how to regulate the aspects associated with vulnerability during adolescence may be a very interesting and important perspective for future theoretical and experimental research.

## Author Contributions

RM wrote the first draft of the manuscript. CL wrote the final version of the manuscript. All authors approved the submitted version.

## Conflict of Interest

The authors declare that the research was conducted in the absence of any commercial or financial relationships that could be construed as a potential conflict of interest.
